# Real-Time Programmable Nonlinear Wavefront Shaping with Si Metasurface Driven by Genetic Algorithm

**DOI:** 10.1016/j.eng.2025.04.023

**Published:** 2025-06

**Authors:** Ze Zheng, Gabriel Sanderson, Soheil Sotoodeh, Chris Clifton, Cuifeng Ying, Mohsen Rahmani, Lei Xu

**Affiliations:** a Advanced Optics and Photonics Laboratory, Department of Engineering, School of Science & Technology, Nottingham Trent University, Nottingham NG11 8NS, UK; b Faculty of Engineering, University of Nottingham, Nottingham NG7 2RD, UK; c Sony Europe B.V., Basingstoke, Hampshire RG22 4SB, UK

**Keywords:** Nonlinear metasurface, Genetic algorithm, Wavefront manipulation

## Abstract

Nonlinear wavefront shaping is crucial for advancing optical technologies, enabling applications in optical computation, information processing, and imaging. However, a significant challenge is that once a metasurface is fabricated, the nonlinear wavefront it generates is fixed, offering little flexibility. This limitation often necessitates the fabrication of different metasurfaces for different wavefronts, which is both time-consuming and inefficient. To address this, we combine evolutionary algorithms with spatial light modulators (SLMs) to dynamically control wavefronts using a single metasurface, reducing the need for multiple fabrications and enabling the generation of arbitrary nonlinear wavefront patterns without requiring complicated optical alignment. We demonstrate this approach by introducing a genetic algorithm (GA) to manipulate visible wavefronts converted from near-infrared light via third-harmonic generation (THG) in a silicon metasurface. The Si metasurface supports multipolar Mie resonances that strongly enhance light-matter interactions, thereby significantly boosting THG emission at resonant positions. Additionally, the cubic relationship between THG emission and the infrared input reduces noise in the diffractive patterns produced by the SLM. This allows for precise experimental engineering of the nonlinear emission patterns with fewer alignment constraints. Our approach paves the way for self-optimized nonlinear wavefront shaping, advancing optical computation and information processing techniques.

## 1 Introduction

Nonlinear metasurfaces, composed of arrays of nano-atoms, have been recognized as a super-compact and versatile platform for nonlinear generation processes, including second-harmonic generation (SHG), third-harmonic generation (THG), sum frequency generation (SFG), four-wave mixing (FWM), and high-order harmonic generation (HHG) [[1], [2], [3], [4], [5], [6], [7], [8], [9], [10], [11]]. Light can be strongly confined within nonlinear metasurfaces with the excitation of multipolar resonances and plasmonics, hence efficiently enhancing the nonlinear generation [12,13]. Particularly, by precisely designing the geometric parameters of nonlinear metasurfaces, the resonant properties can be manipulated and optimized, enabling flexible control over the amplitude, phase, and polarization of the nonlinear emission [14]. Therefore, nonlinear metasurfaces have been widely applied in various fields, including nonlinear imaging [11,15,16], quantum light sources [[17], [18], [19], [20]], ultrasensitive sensing [21,22], and optical computation [23]. However, the tunable and automatic engineering of nonlinear wavefronts generated by metasurfaces remains a significant challenge, attracting considerable attention in the field [24,25].

Genetic algorithm (GA), inspired by the process of natural selection, is a metaheuristic optimization algorithm in computer science [26]. Via utilizing the biologically inspired operators, such as mutation, crossover, and selection, solutions or parameters can be automatically searched and optimized in computable systems, where the cost or fitness functions can be applied. Thanks to these capabilities, GA has been extensively employed in the design of conventional and integrated optical components, including lenses [27], filters [28,29], waveguides [[30], [31], [32]], and metasurfaces [33]. Another key application of GA in optics is its integration with multipixel amplitude or phase modulation devices, such as spatial light modulators (SLMs) and digital micromirror devices (DMDs), for wavefront shaping in applications like holography and multi-point focusing through scattering media [[34], [35], [36], [37]]. However, the diffraction effects inherent in multipixel devices can introduce errors and noise into the signal collection. To address this, it is often necessary to mitigate diffraction through the use of spatial filters or advanced denoising techniques.

Optical data processing has gained prominence in recent years as an innovative approach, offering faster response times, lower energy consumption, and a smaller footprint compared to traditional electronic signal processing [23,[38], [39], [40], [41], [42]]. Concurrently, metasurfaces have emerged as a promising platform for optical computing, thanks to their compactness and versatility [23,38,41,42]. By carefully designing their resonant properties, metasurfaces can enable precise control of spatial amplitude and phase in free space, along with unique interactions in momentum space. Furthermore, nonlinear metasurfaces expand beyond the limitations of linear optics, opening up new possibilities for configuring quadratic, cubic, and even higher-order responses as operators in data processing. These nonlinear responses can be further engineered in the momentum space, enabling the functionalities of metasurfaces in different image processing applications [23].

Here, we experimentally develop an optical system to manipulate the nonlinear wavefront in free space by utilizing the SLM, GA, and Si metasurface. The GA optimizes the phase distribution of the SLM, allowing the nonlinear wavefront to be shaped according to input reference images. The third-harmonic generation (THG) emission is significantly enhanced through multipolar resonances, enabling efficient nonlinear conversion. Furthermore, the cubic relationship of the THG in the Si metasurface acts as a low-signal filter, suppressing diffraction signals from the SLM and serving as an effective denoising operator. Our proposed system eliminates the need to position the SLM in the Fourier plane, increasing tolerance to optical alignment errors, and offering a promising example for next-generation optical data processing and self-aligned devices.

## 2 Results

**Fig. 1:**
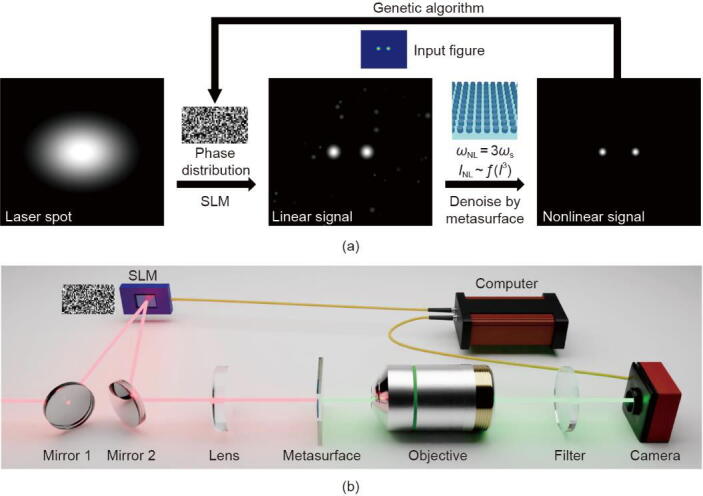
(a) Schematic illustration of the proposed signal modulation process. Left: initial input laser spot illustration before interacting with the SLM and Si metasurface. Middle: schematic of the linear signal image diffracted by the SLM. Right: schematic of the nonlinear signal image generated by the Si metasurface. $\omega_{NL}$ is the nonlinear wavelength (third harmonic wavelength), $\omega_{s}$ is the signal wavelength, and $I_{NL}$ is the optical intensity distribution of the nonlinear emission detected by the camera. The function $f(I^{3})$ represents the nonlinear resonant response of the silicon metasurface, exhibiting a dependence on the cube of the input intensity distribution of the signal beam. (b) The schematic diagram of the experimental setup for the proposed optical system.

Here we use Fig. 1(a) to illustrate the conversion process from an infrared input laser beam into the expected nonlinear wavefront. First, a laser spot is diffracted by the SLM, transforming it into the linear signal, which is a signal that maintains a proportional relationship to the input without altering its frequency components. This linear signal is then converted and denoised by the Si metasurface through a nonlinear optical process, generating a nonlinear signal. Unlike the linear signal, the nonlinear signal includes frequency-shifted components produced by the interaction with the metasurface. Our approach enables the generation of a clear two-point pattern of the nonlinear signal, closely matching the input figure. More specifically, two arrows used in Fig. 1(a) represent two important procedures in our optical system. The first arrow, labeled “SLM and GA”, involves utilizing the reflective SLM to manipulate the phase distribution of the laser beam. The phase distribution is optimized using the GA based on the input figure which is the expected nonlinear emission. The GA evolves based on the captured nonlinear emission from the charge-coupled device (CCD) camera, so that a real-time response in the GA can be established. It can be seen in Fig. 1(a) that after the SLM the laser spot is manipulated into the linear signal with two big light points in the center according to the input figure. However, diffraction from the SLM may produce small, undesired spots as noise. Fortunately, the second procedure, indicated by the other arrow, can not only convert the infrared signal into visible light but also help to filter out those undesirable small spots based on the THG process, as illustrated in Fig. 1(a). The cubic response between the linear and nonlinear signals allows the Si metasurface to act as a denoising optical processor for the linear signal. With other nonlinear effects, such as second-order nonlinear effects, we believe denoising can still operate in a nonlinear relation. However, the crystal orientation of second-order nonlinear materials may introduce polarization dependence into the denoising process, rather than relying solely on intensity-based filtering. Additionally, please note that the GA-based optimization process of the nonlinear wavefront is dynamic. Users can reset the input figure during the evolution process, allowing the wavefront to be optimized generation by generation, approaching the new target. However, forming more complex wavefronts requires a longer evolution time.

Fig. 1(b) gives the experimental optical setup we used to shape the nonlinear wavefront. A femtosecond laser beam with a central wavelength of 1510 nm is reflected from Mirror 1 to the SLM with the real-time optimizing phase distribution. The angle is around 10 degrees between the incident direction of the laser beam and the normal direction of the SLM. Hence, the laser beam profile is modulated and reflected to the Mirror 2, then collected by the lens, and focused onto the Si metasurface. The converted nonlinear signal from the Si metasurface is collected by the objective and imaged with a visible camera. A 600 nm short-pass filter is used for observing only the converted THG signal. The camera is connected to the computer, so the real-time wavefront information can be transferred to the GA. And the GA then adjusts the phase distribution of the SLM, according to the captured images from the camera. The detailed information on each experimental component is included in the Section S1 in Appendix A.

**Fig. 2:**
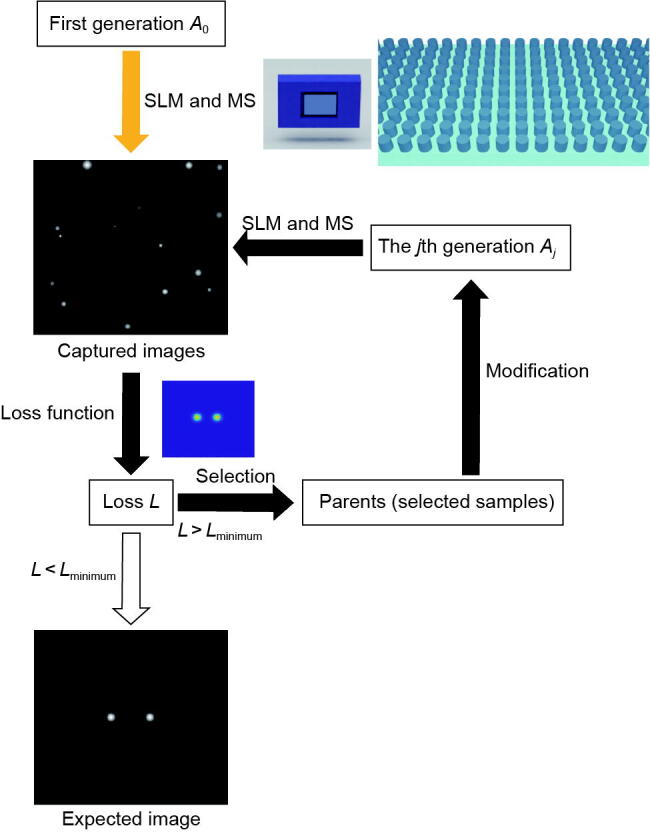
The flowchart of GA employed in the optical system with the metasurface (MS). $A_{0}$ and $A_{j}$ denote the initial population and the evolved population at the *j*th generation, respectively, each consisting of 50 SLM phase distributions used in the evaluation process. L is the loss function and $L_{minimum}$ is the target loss function criterion.

During this process, the SLM and the camera are connected to the computer. We employ the GA optimization method, which is an iterative numerical optimization method based on stochastic global search with the flowchart shown in Fig. 2. We implement our algorithm via MATLAB (The MathWorks Inc., USA). The input to the algorithm is the experimentally measured nonlinear emission intensity pattern from the CCD camera, with the goal of matching it to a predefined target pattern (the input image). For simplicity, the population size is set to 50. First, our GA program specifies 50 random SLM phase distributions, and accordingly, obtains an initial population of 50 experimentally measured nonlinear emission intensity patterns from the CCD. Each measured nonlinear emission pattern is then evaluated using the loss function *L*. The loss function is defined as $L=\sum_{i=1}^{m}\sum_{k=1}^{n}\left(a_{\mathrm{ik}}^{\mathrm{LN}}-a_{\mathrm{ik}}^{input}\right)$, where $a_{\mathrm{ik}}^{\mathrm{LN}}$ and $a_{\mathrm{ik}}^{input}$ are the pixel value in the *i*th row and *k*th column of the captured image and the input image, respectively. *m* and *n* represent the number of rows and columns of the selected pixel array in the CCD camera, respectively. The product *m* × *n* corresponds to the total number of pixels in the captured 2D intensity distribution. The loss function is the key index to evaluate the difference between the input and generated patterns. Here we linearly normalize the pixel values of the captured image, taking the highest pixel value as 255. With a predefined mutation rate of 0.15, the top 25 patterns are selected for mutation and breeding to generate the next generation of phase distributions required by the SLM. The GA program will continue to run until the target loss function criterion ($L_{minimum}$) is met. Our GA optimization method enables real-time self-correction of generated patterns. Even if misalignments or shifts occur in the optical components, the GA facilitates phase distribution evolution until a new stable distribution is achieved. However, the evolutionary process limits real-time adaptation to highly complex pattern changes. Neural networks are recognized as advanced algorithms capable of significantly increasing processing speed when properly trained [43]. We believe that integrating neural networks with our optical system can greatly reduce processing time.

**Fig. 3:**
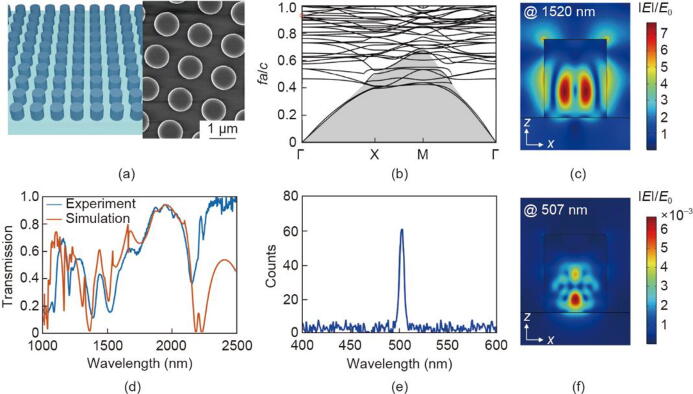
(a) Left: the schematic diagram of the Si metasurface. Right: SEM images of the fabricated Si metasurface. (b) The calculated band structure of the Si metasurface. The frequency is normalized as fa/c, where f represents the optical frequency, *a* is the periodicity of the metasurface, and c is the speed of light in vacuum. X, M, and Γ are high-symmetry points in the Brillouin zone of the band structure. (c) The simulated electric field distribution at the resonant position of 1520 nm. The normalized electric field intensity $\left|E\right|/E_{0}$ is used to evaluate local field enhancement within the metasurface. Here, $E_{0}$ is defined as the amplitude of the incident plane wave. (d) The simulated and measured transmission spectra of the Si metasurface at normal incidence. (e) The measured THG emission spectrum from Si metasurface under the illumination of the femtosecond laser at 1510 nm. (f) The simulated field distribution of generated nonlinear emission at around 507 nm.

We then demonstrate the properties of the Si metasurface (Fig. 3) consisting of the square array of Si nanodisks on top of a SiO_2_ substrate, as depicted in Fig. 3(a). The height of the nanodisks is H = 1000 nm. The radius of the nanodisks is $r_{0}$ = 400 nm. The periodicity of the metasurface is 1400 nm in *x* and *y* directions. We simulate the linear and nonlinear optical properties of the Si metasurface with the refractive indices of Si and SiO_2_ as 3.4 and 1.5, respectively. By calculating the band structure of the Si metasurface, the resonant dispersion in the k-space is obtained, as shown in Fig. 3(b). It can be seen that a flat band structure is observed, predicting a minimal resonant shift in the k-space. The momentum dispersion of selected resonances affects the filtering of spatial frequencies in the image conversion process. For the utilized resonance (red circle), a relatively flat band structure is observed, suggesting that most spatial frequencies can be preserved during the nonlinear generation process. By designing the metasurfaces with different resonances like multipolar Mie resonances, toroidal dipole resonances, and guided-mode resonances, the band structures with different behaviors can be achieved. In this way, the specific response to the spatial frequencies of the image can be processed with the carefully designed metasurface. In this paper, we focus on detecting the nonlinear spots with strong amplitudes at the expected positions and filtering out the weak spots, so that a flat band structure helps make our metasurface less sensitive to the spatial frequencies of the image after being modulated by the SLM.

The calculated multipolar analysis shows the resonance at around 1520 nm is mainly governed by magnetic dipole (MD), demonstrated in Section S2 in Appendix A [[44], [45], [46]]. Fig. 3(d) shows the measured and simulated transmission spectra, which agree well with each other. Here, we use the MD resonance at around 1520 nm to further enhance the THG emission from the Si metasurface. The discrepancy between the simulated (1520 nm) and measured (1510 nm) resonant positions arises from the fabrication inconsistencies. The electric field distribution is depicted in Fig. 3(c). A strong field enhancement inside the Si nanodisks is achieved, then significantly enhancing the THG emission following the frequency relation $\omega_{THG}=3\omega_{signal}$, where $\omega_{THG}$ and $\omega_{signal}$ are the wavelengths of the nonlinear emission and input signal beam, respectively. As a result, a THG emission at around 507 nm is generated as shown in Fig. 3(e) with the field distribution in Fig. 3(f) [[47], [48], [49], [50]]. The detailed information regarding the nonlinear interactions within the Si metasurface can be found in the Section S3 in Appendix A. The field distributions of the electric displacements are shown in Section S4 in Appendix A. The forward THG conversion efficiency is measured, illustrated in Section S5 in Appendix A. In general, the efficiency of nonlinear processes is lower than that of linear processes. However, we believe our technique introduces new possibilities for implementing nonlinear operators in image processing and optical computing.

**Fig. 4:**
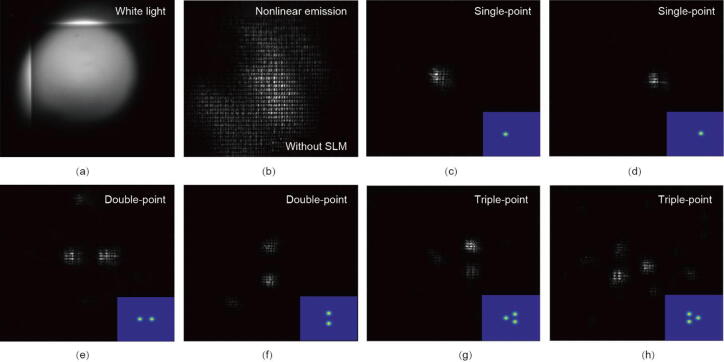
(a) The measured images of the Si metasurface under white light source illumination. (b) The nonlinear emission pattern with the flat phase distribution of the SLM (without the modulation of the SLM) (c–h) The modulated nonlinear emission pattern via the GA based on the input figure shown at the bottom, demonstrating the single focusing point at (c) left and (d) right, the double focusing points with (e) horizontal and (f) vertical arrangement, and the triple focusing points with arrow patterns toward (g) left and (h) right.

The experimental results of our proposed optical system are demonstrated in Fig. 4. The nonlinear emission after the SLM with the flat phase distribution is shown in Fig. 4(b), where the SLM acts as a mirror without any modulation effect on the wavefront. Then the reference images with different patterns are input into the GA, and then the phase distribution of the SLM is optimized in real-time, generation by generation, according to the nonlinear pattern captured by the CCD camera (as shown in Fig. 1, Fig. 2). For single-point cases (Figs. 4(c) and (d)), a clear focusing point at the expected position is observed after evolving through around 100 generations in the GA (approximately an hour). The number of generations and time for evolving based on the GA increase when having the input figures with more complex patterns. For double-point cases (Figs. 4(e) and (f)), the number of generations for observing the clear double-focusing pattern is around 200 (approximately two hours). For triple-point cases (Figs. 4(g) and (h)), the number of generations would reach around 400 (approximately four hours). Since the modification process in the GA is stochastic, the number of generations for different patterns is estimated based on the different experimental configurations. The discrepancy between the input and generated patterns is influenced by the choice of the loss function criterion. We believe the fidelity of the generated patterns can be further enhanced by optimizing the loss function, allowing for longer evolution time, and utilizing a spatial light modulator with a higher filling factor and improved resolution. In conclusion, we operate the real-time modulation of the nonlinear wavefront with different focusing patterns, the number of focusing points and the positions of each point can be precisely controlled. We believe our approach has potential for various experimental applications, one of which is single-pixel imaging. Single-pixel imaging is an imaging technique that reconstructs images using a single-pixel photodetector [51]. This process relies on a sequence of structured wavefronts being illuminated onto the object. Our nonlinear wavefront shaping system is well-suited for single-pixel imaging, enabling a nonlinear and self-correcting imaging approach.

The detailed information regarding the materials and methods of the numerical simulations [52], metasurface fabrication, and experiment is presented in the Section S1.

## 3 Discussion and summary

In this paper, we demonstrate real-time programmable nonlinear wavefront shaping with the Si metasurface by connecting the SLM and the CCD camera via the GA. The GA can optimize the phase distribution of the SLM according to the real-time captured nonlinear images generation by generation until the nonlinear pattern matches the input image. Via designing a resonance at 1510 nm, an efficient THG conversion is achieved via the Si metasurface, so that the infrared beam can be transformed into visible light after the modulation of the SLM. Moreover, the cubic response of the THG enables the Si metasurface to perform a denoising operation on the wavefront, so that the undesired weak noise can be filtered out. Our results hold promising potential for advancing the real-time nonlinear wavefront shaping of metasurfaces, expanding applications in optical computation, and information processing and imaging based on nonlinear metasurfaces.

## CRediT authorship contribution statement

**Ze Zheng:** Writing – review & editing, Writing – original draft, Validation, Software, Methodology, Investigation, Conceptualization. **Gabriel Sanderson:** Writing – review & editing, Investigation. **Soheil Sotoodeh:** Writing – review & editing, Resources. **Chris Clifton:** Writing – review & editing, Resources. **Cuifeng Ying:** Writing – review & editing, Supervision, Investigation. **Mohsen Rahmani:** Writing – review & editing, Validation, Supervision, Resources, Conceptualization. **Lei Xu:** Supervision, Project administration, Conceptualization.

## Declaration of competing interest

The authors declare that they have no known competing financial interests or personal relationships that could have appeared to influence the work reported in this paper.
