# Supplementary material for: Real-Time Programmable Nonlinear Wavefront Shaping with Si Metasurface Driven by Genetic Algorithm
Source: Engineering (Beijing). 2025 Jun;49:90–5. doi: 10.1016/j.eng.2025.04.023 (PMC12239666; doi:10.1016/j.eng.2025.04.023)
Supplement: Supplementary Data 1 [file mmc1.docx]

Supplement Materials

**Real-Time Programmable Nonlinear Wavefront Shaping with Si Metasurface Driven by Genetic Algorithm**

Ze Zheng ^a^, Gabriel Sanderson ^a,b^, Soheil Sotoodeh ^c^, Chris Clifton ^c^, Cuifeng Ying ^a^, Mohsen Rahmani ^a^, Lei Xu ^a^

^a^ *Advanced Optics and Photonics Laboratory, Department of Engineering, School of Science & Technology, Nottingham Trent University, Nottingham, NG11 8NS, UK*

^b^ *Faculty of Engineering, University of Nottingham, Nottingham NG7 2RD, UK*

^c^ *Sony Europe B.V., Basingstoke, Hampshire, RG22 4SB, UK*

* Corresponding authors.

*E-mail addresses:* mohsen.rahmani@ntu.ac.uk (M. Rahmani), lei.xu@ntu.ac.uk (L. Xu).

**OUTLINE:**

1. Materials and Methods

2. The Multipolar Analysis

3. Nonlinear Interation between Input and Nonlinear Wavefronts

4. The field Distributions of the Electric Displacement

5. Conversion Efficiency of the Third-harmonic Generation

6. References

**1. Materials and Methods**

**Numerical simulations**

The field patterns, transmittance spectra, and multipolar analysis are calculated using the finite element method (FEM) in COMSOL Multiphysics 6.2 software. The calculation of the band structure is based on the Massachusetts Institute of Technology Photonic-Bands (MPB) open sources[5]. Figure 1(a) is created based on Blender 3.1.0 using an optical components pack provided by Ryo Mizuta Graphics.

**Metasurface fabrication**

We first used plasma-enhanced chemical vapor deposition to deposit an amorphous silicon layer on the quartz substrate. Meanwhile, a resist layer (ZEP520) is spin-coated on the wafer. The periodic patterns were then defined in the resist layer with electron-beam-lithography. Using the resist as a mask layer, the patterns were transferred to the silicon diaphragm by the inductively coupled plasma technique. Finally, the remaining resist was removed with the Nmethyl-2-pyrrolidone (NMP) liquor.

**Experiments**

The femtosecond laser beam with 150 fs pulse duration and an 80 MHz repetition rate at the central wavelength of 1510 nm is used in experiments. The SLM is from Holoeye (LUNA-TELCO-115) with a pixel pitch of 4.5 µm, a resolution of 1920 × 1080, and an active area of 8.64 × 4.86 mm. In our experiments, we set every 40 × 40 pixels as one unit to modulate the phase distribution. The resulting image was captured by a 20× objective with NA=0.4 and detected using a CCD camera (CS165MU/M, Thorlabs) and spectrometer (Ocean Insight, QEPRO-XR) for measuring emission intensity.

**2. The Multipolar Analysis**

We analyze the behaviors of these five resonances by performing the spherical multipolar analysis demonstrated in Fig. S1. The ED, MD, EQ, MQ, EO, MO respectively represent the electric dipole (ED) and magnetic dipole (MD), electric quadrupole (EQ) and magnetic quadrupole (MQ), electric octupole (EO) and magnetic octupole (MO).


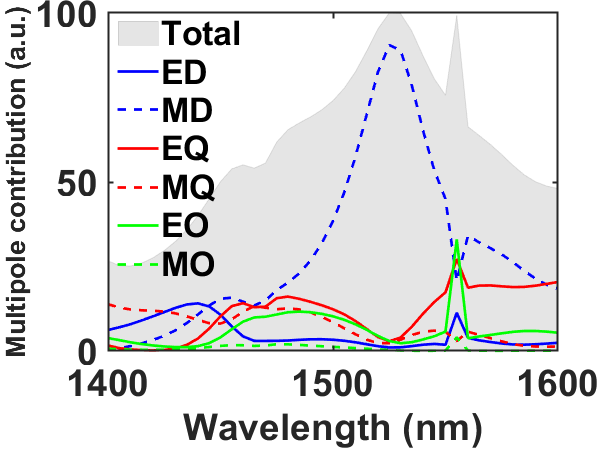


Figure S1. The calculated multipolar analysis of the Si metasurface from 1400 to 1600 nm.

**3. The Nonlinear Interation between Input and Nonlinear Wavefronts**

The nonlinear responses of the Si metasurface were modeled numerically using the finite-element method in COMSOL Multiphysics in the frequency domain. We assumed the undepleted pump field approximation and followed two steps to model the nonlinear response [1,2]. The electric field at the pump and signal beam were simulated first, and the nonlinear polarization induced inside the Si disk-film metasurface was obtained. We then employed the obtained nonlinear polarization as a source for the electromagnetic simulation at the harmonic wavelength to obtain the generated THG field. The induced nonlinear polarization component $\mathbf{P}^{\mathrm{THG}}$ can be simplified as $\mathbf{P}^{\mathrm{THG}}=\varepsilon_{0} \chi^{(3)}(\mathbf{E}\cdot\mathbf{E})\mathbf{E}$, where $\varepsilon_{0}$ is the permittivity of free space, $\chi^{(3)}$ is the third-order nonlinear susceptibility of silicon, $\mathbf{E}$ represents the electric field of the incident light. Figure 3(f) is simulated under the plane-wave incident with the power intensity 0.15 GW/cm^2^ [3,4]. The third-order nonlinear susceptibility of silicon is set as $\chi^{(3)}=2.45\times{10}^{-19} m^{2}V^{-2}$. For the incident light at Gamma point in the momentum space, the nonlinear interaction behaves as a third-order intensity filter, described by: $I^{\mathrm{PL}}=\alpha I^{3}$, where *α* is the normalized nonlinear coefficient, and *I* is the intensity of the input light.

**4. Field Distributions of the Electric Displacement**


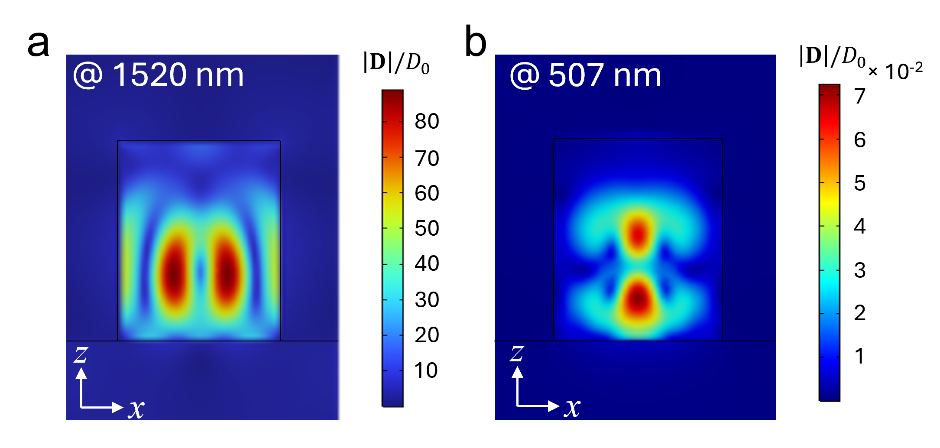


Figure S2. The calculated linear (a) and nonlinear (b) field distributions of the electric displacement of the Si metasurface. $D_{0}$ is the electric displacement field amplitude of the incident plane wave in vacumm.

**5. The Conversion Efficiency of the Third-harmonic Generation**

We measured the conversion efficiency of the Si metasurface under the femtosecond laser with the wavelength of 1510 nm. The laser is focused onto the metasurface sample by a 10× objective with NA = 0.26 with a spot size of around 20 µm. A 100× objective with NA = 0.7 is utilized to collect the THG emissions. The laser input power is 56 mW. As a result, we have observed the forward THG emission with the power of 7.7 nW. The conversion efficiencies ($\eta_{\mathrm{THG}}$ and ${\eta'}_{\mathrm{THG}}$) with different difinations can be calculated based on the equations: $\eta_{\mathrm{THG}}= P_{\mathrm{THG}}/P_{\mathrm{input}}^{3}$ and ${\eta'}_{\mathrm{THG}}= P_{\mathrm{THG}}/P_{\mathrm{input}}$, where $P_{\mathrm{THG}}$ and $P_{\mathrm{input}}$ are respectively the measured power of the forward THG emission and input beam. We obtained the conversion efficiencies $\eta_{\mathrm{THG}}=$ 4.385 × 10^-5^ W^-2^ and ${\eta'}_{\mathrm{THG}}=$ 1.375 × 10^-7^. Our method introduces a dynamic optimization of the nonlinear wavefront, employing GA and SLM to provide improved and flexible control, compared with the traditional Pancharatnam-Berry phase approach.

**6. Refernces**

[1] L. Carletti, A. Locatelli, O. Stepanenko, G. Leo, C. De Angelis, Enhanced second-harmonic generation from magnetic resonance in algaas nanoantennas, Optics Express 23 (20) (2015) 26544-26550.

[2] D. A. Smirnova, A. B. Khanikaev, L. A. Smirnov, Y. S. Kivshar, Multipolar third-harmonic generation driven by optically induced magnetic resonances, ACS Photonics 3 (8) (2016) 1468-1476.

[3] D. Moss, E. Ghahramani, J. Sipe, H. Van Driel, Band-structure calculation of dispersion and anisotropy in χ → (3) for third-harmonic generation in si, ge, and gaas, Physical Review B 41 (3) (1990) 1542.

[4] E. Dulkeith, Y. A. Vlasov, X. Chen, N. C. Panoiu, R. M. Osgood Jr, Self-phase-modulation in submicron silicon-on-insulator photonic wires, Optics Express 14 (12) (2006) 5524-5534.

[5] S. G. Johnson, J. D. Joannopoulos, Block-iterative frequency-domain methods for maxwell’s equations in a planewave basis, Optics Express 8 (3) (2001) 173–190.
